# Pancreatic cancer-derived exosomes transfer miRNAs to dendritic cells and inhibit RFXAP expression via miR-212-3p

**DOI:** 10.18632/oncotarget.4924

**Published:** 2015-08-14

**Authors:** Guoping Ding, Liangjing Zhou, Yingming Qian, Mingnian Fu, Jian Chen, Jionghuang Chen, Jianyang Xiang, Zhengrong Wu, Guixing Jiang, Liping Cao

**Affiliations:** ^1^ Department of General Surgery, Sir Run Run Shaw Hospital, School of Medicine, Zhejiang University, Hangzhou, China

**Keywords:** pancreatic cancer, exosomes, RFXAP, MHC II, miR-212-3p

## Abstract

It has been reported tumor-derived exosomes can transfer miRNAs to recipient cells in the tumor microenvironment, promoting tumor invasion and metastasis. The present research aimed to explore how pancreatic cancer (PC) derived exosomal miRNAs inhibited mRNA expression of dendritic cells and induced immune tolerance. Our study revealed that 9 PC-related miRNAs were increased and 208 mRNAs were inhibited in exosome-stimulated dendritic cells (exo-iDCs) compared to immature dendritic cells (iDCs). A target prediction between the 9 miRNAs and 208 mRNAs was performed by bioinformatics database analysis. From the target prediction, it was predicted and validated that regulatory factor X-associated protein (RFXAP), an important transcription factor for MHC II, was inhibited by miR-212-3p transferred from PC-secreted exosomes, resulting in decreased MHC II expression. Moreover, a clinical study showed a negative correlation between miR-212-3p and RFXAP in PC tissue. From these data, we concluded that PC-related miRNAs can be transferred to dendritic cells via exosome and inhibit target mRNA expression. More importantly, PC-derived exosomes inhibit RFXAP expression via miR-212-3p, which decrease MHC II expression and induce immune tolerance of dendritic cells. RFXAP deficiency has never been reported in solid tumors. The functions and mechanisms of RFXAP in tumors deserve future explorations.

## INTRODUCTION

Pancreatic cancer (PC) is one of the most malignant cancers, with a 5-year survival rate of less than 5% [1]. In 2014, there were 39 590 patients who died of pancreatic adenocarcinoma in the United States, making it the fourth leading cause of cancer-related death [2]. The prognosis of PC is extremely poor, as more than 80% of patients are diagnosed at an advanced stage and are no longer eligible for surgical resection because of vessel invasion or distant metastasis. Even for patients who have undergone radical surgery, the 5-year survival rate is only 8.47%, and the rate of recurrence within 1 year is up to 54% [3]. Pancreatic cancer is characterized by strong invasion of tissue and metastasis [4, 5], while the mechanism involved has not yet been fully elucidated. A better understanding of the molecular pathogenesis of PC metastasis would be helpful for early detection and the development of new therapeutic strategies against PC.

Exosomes are small extracellular vesicles approximately 30–110 nm in size. They are secreted by a wide variety of cell types including tumor cells [6, 7]. Our previous study demonstrated that PC-derived exosomes inhibit TLR4 expression of dendritic cells (DCs), inducing immune tolerance [8]. However, PC-derived exosomal miRNAs and their function in antigen-presenting cells are still poorly understood. Therefore, one purpose of the current study was to confirm the transfer of PC miRNAs into dendritic cells by exosomes and construct a regulatory network between PC-related miRNAs and DC mRNAs, which would help to find new target genes in DCs that are regulated by PC-derived miRNAs.

Regulatory factor X-associated protein (RFXAP) is a key transcription factor for the MHC II gene, and its deficiency can lead to a rare severe immunodeficiency disorder termed bare lymphocyte syndrome [9, 10]. Down-regulation of RFXAP expression can inhibit MHC class II expression, leading to inactivation of CD4^+^ T-lymphocyte [11]. However, few studies have investigated the role of RFXAP deficiency in tumor progression. In our study, mRNA chip and western blot analysis indicated that RFXAP was inhibited in PC exosome-stimulated dendritic cells. Thus, it is especially important to clarify the mechanism by which PC-derived exosomal miRNAs regulate RFXAP expression in dendritic cells.

## RESULTS

### Characterization of tumor exosomes and iDCs

**Figure 1:**
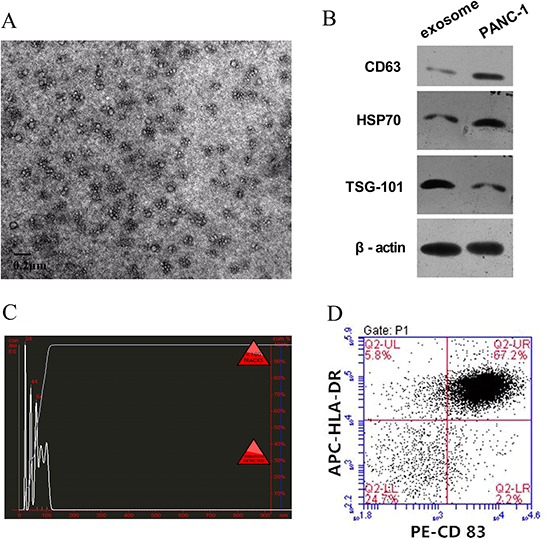
Characterize of tumor exosomes and iDC **A.** Exosomes observed by electron microscopy, which showed typical vesicular structures. **B.** Western blot analysis of PANC-1 derived exosomes. CD63, HSP70, and TSG101 could be detected in PANC-1 derived exosomes and PANC-1 cells. **C.** By NanoSight particle-tracking analysis, the size of exosomes distributed in the range from 30 nm to 110 nm. **D.** iDC was confirmed by flow cytometry. The double positive rate of CD83 and HLA-DR in the iDC attained up to 67.2%.

Exosomes were collected from tumor cell culture media by ultracentrifugation Their identity was confirmed by electron microscopy (Figure 1A) and the presence of CD63, HSP70 and TSG101, known exosome specific markers (Figure 1B). Size distribution analysis was performed with a Nanosight NS300, and all the particles were in the range of 30–110 nm (Figure 1C).

Immature DCs (iDCs) were induced from peripheral blood monocytes and cultured for up to 7 days. Confirmation was made by staining with antibodies to CD83 and HLA-DR followed by flow cytometry. The percentage of CD83^+^ HLA-DR^+^ cells attained was up to 67.2% (Figure 1D).

### PC-derived miRNAs were transferred into iDCs via exosome

**Figure 2:**
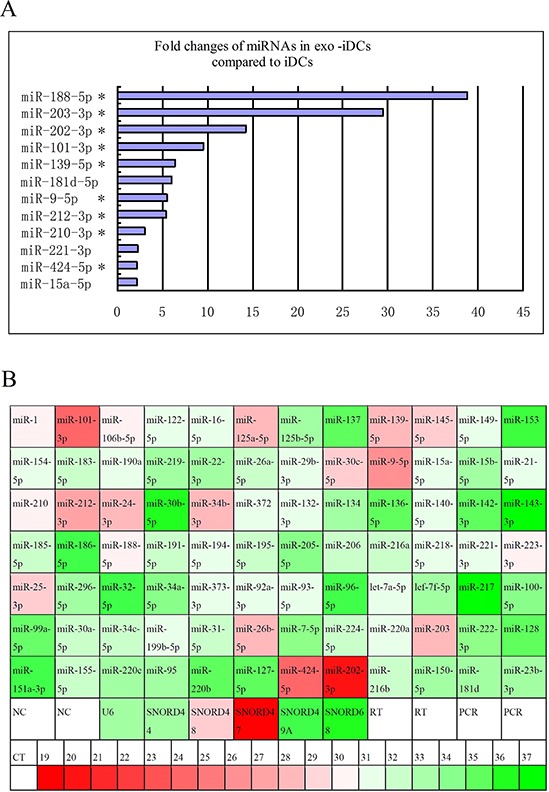
Exosome transfer PC-derived miRNAs to iDCs **A.** Fold change of miRs in exosome-stimulated DCs compared to untreated DCs, 12 PC-related miRNAs were increased more than 2-fold in exo-iDCs compared with iDCs based on miRNA qPCR array analysis. * indicate that miRNAs were also highly expressed in PANC-1 exosomes. **B.** The expression profiles of 84 known PC related miRNA in PANC-1 derived exosomes. 19 miRNAs were highly expressed in PANC-1 derived exosomes (CT ≤ 30). The CT value is marked with color, green indicates low relative expression and red indicates high expression.

miRNA qPCR arrays containing 84 known PC-related miRNAs were used to identify differentially expressed miRNAs in PANC-1-derived exosomes, iDCs and exosome-stimulated iDCs. There were 12 PC-related miRNAs increased more than 2-fold in exo-iDCs compared with iDCs (Figure 2A), among which 9 miRNAs were also highly expressed in PANC-1-derived exosomes (Figure 2B). This indicates that PC-derived exosomes can transfer miRNAs to immature dendritic cells.

### mRNA profile of DCs and miRNA target prediction

**Figure 3:**
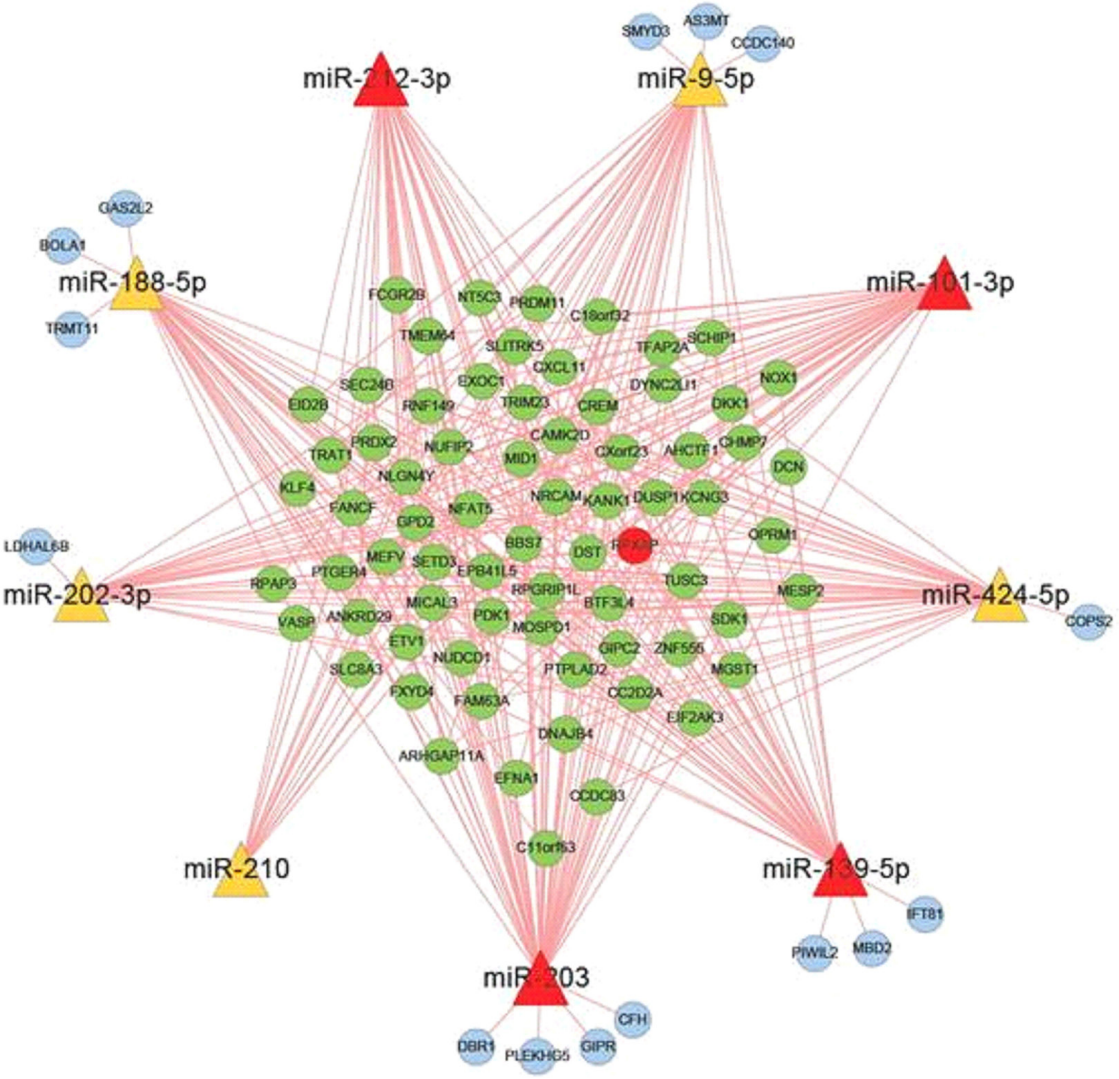
Target prediction between PC related miRNA and DC mRNA The miRNA target prediction between 9 PC derived exosomal miRNAs and 208 down-regulated mRNA in dendritic cells. miR-203, miR-101-3p, miR-212-3p and miR-139-5p are the potential regulators that can inhibite RFXAP expression.

All RNA samples that were used for miRNA examination were also used for the mRNA chip analysis, so that the two data sets could be matched. The results showed that a total of 5 097 mRNAs were increased and 4 929 mRNAs were decreased in exo-iDCs compared with iDCs. All raw data has been deposited in the GEO Database (http://www.ncbi.nlm.nih.gov/geo/) under accession number GSE67020. Among the mRNAs of interest, 208 were negatively expressed in exo-iDC with an absolute change greater than four-fold compared to iDC (Supplementary Table S1). The 208 negatively expressed mRNAs were considered as candidate target mRNAs, which may be inhibited by exosomal miRNAs transferred from PANC-1 secreted exosomes. For the 208 negatively expressed mRNAs and 9 up-regulated PDAC-related miRNAs, target correlations were predicted by retrieving sequences from databases, and the predicted target network between PC-related miRNAs and DC mRNA was established (Figure 3 and Supplementary Table S2).

### RFXAP expression was inhibited in exo-iDCs

**Figure 4:**
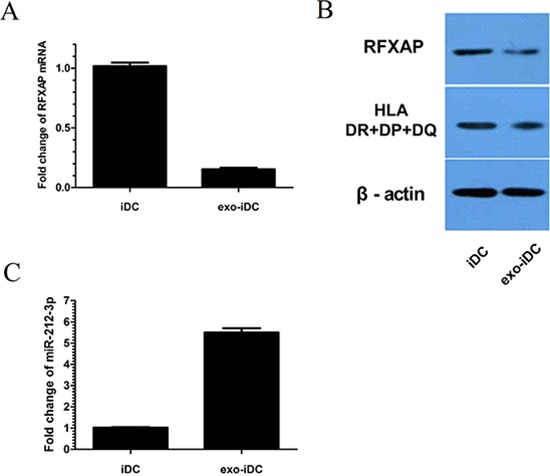
RFXAP was inhibited and miR-212-3p was increased in exo-iDCs **A.** qRT-PCR indicated that RFXAP mRNA declined by 84.6% after stimulation by PANC-1 derived exosomes. **B.** Western blot showed RFXAP and MHC II expression were inhibited in exo-iDC. **C.** There were 5.5 folds changes between iDC and exo-iDC, indicating miR-212-3p was transferred into iDC by exosome.

In the predicted miRNA-mRNA target network, RFXAP was selected out as its deficiency may lead to suppression of MHC II molecules and immune tolerance, which has never been reported in solid tumors. In mRNA chip, RFXAP was negatively expressed in exo-iDCs and decreased by 6.77 folds compared to iDCs (Supplementary Table S1). In the present study, DCs were stimulated with PC derived exosome. qRT-PCR further confirmed RFXAP mRNA was decreased by 84.6% (Figure 4A). Western blot analysis also showed that RFXAP and MHC II molecules were decreased in exo-iDCs (Figure 4B), indicating that RFXAP and MHC II molecules were inhibited by PC-derived exosomes. miR-212-3p was increased by 5.5 folds in exo-iDCs compared to iDCs, similar to the results in figure 2A (Figure 4C).

### PANC-1-derived exosomes inhibited RFXAP and MHC II expression via miR-212-3p

**Figure 5:**
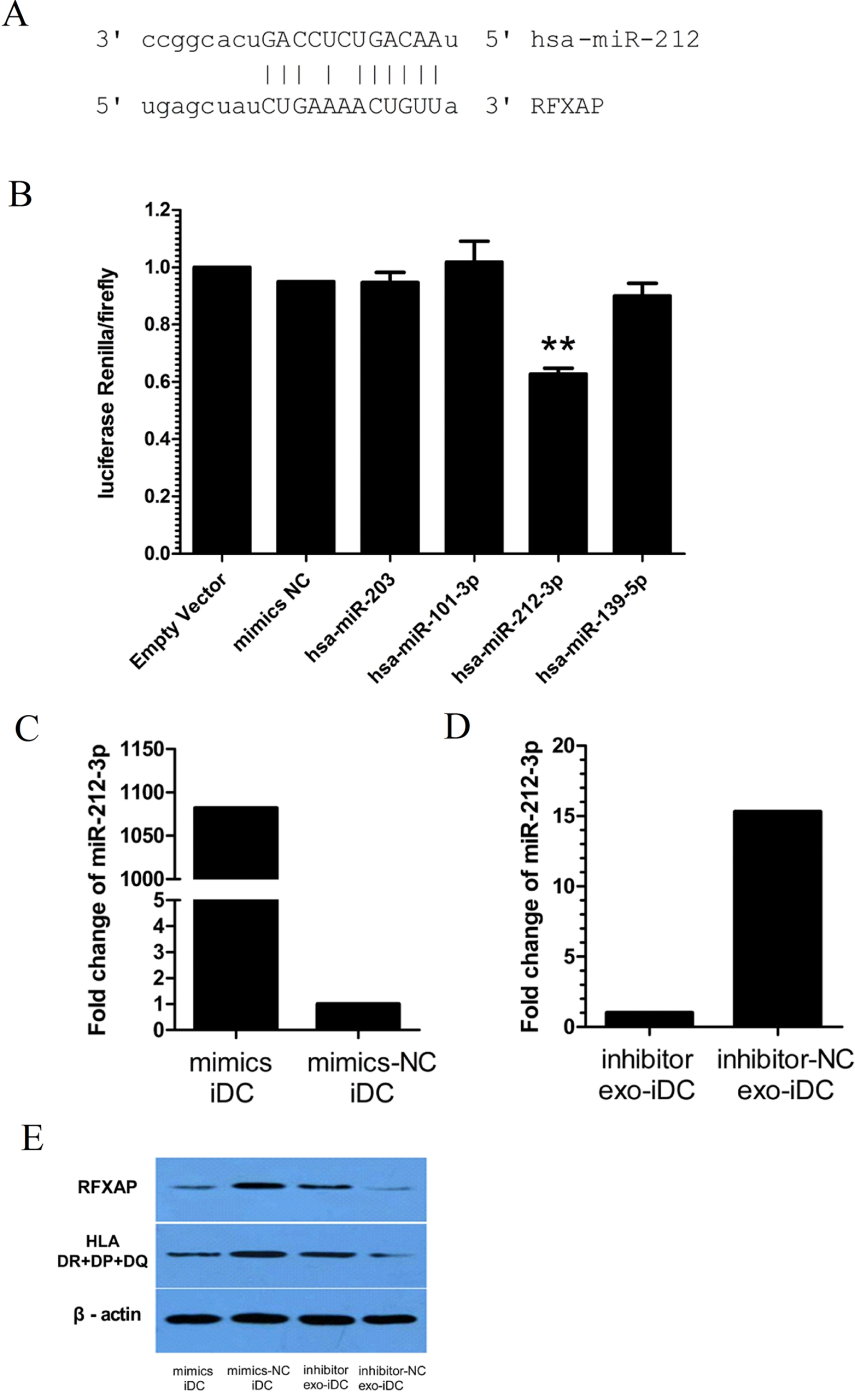
PANC-1-derived exosomes inhibit RFXAP and MHC II expression via miR-212-3p **A.** A panel that described the site of binding between miR-212-3p and RFXAP 3′UTR. **B.** Luciferase activity ratio was determined 48 h after 293T cells were transfected with miRNA mimics or mimics NC. The results showed that miR-212-3p decreased luciferase expression by 37.2%, while the other miRNAs did not. Data are presented as means ± S.E.Ms. ** denotes obvious statistical significance (*p* < 0.01). **C.** miR-212-3p mimics and **D.** inhibitors were transfected into iDCs and exo-iDCs respectively. miR-212-3p was increased 210 folds in iDC after miR-212-3p mimics transfection. miR-212-3p was decreased 23 folds in exo-iDC after miR-212-3p inhibitors transfection. **E.** By Western blot, miR-212-3p mimics transfected iDCs showed decreased RFXAP and MHC II expression compared with mimics NC transfected iDCs. Inhibitors transfected exo-iDCs showed an increased expression of RFXAP and MHC II compared with inhibitor NC transfected exo-iDC. β-actin was used as an internal control.

In the predicted miRNA-mRNA target network, miR-203, miR-101-3p, miR-212-3p, and miR-139-5p were predicted to bind to RFXAP mRNA (Figure 3). Luciferase expression analyses were conducted to validate whether miR-203, miR-101-3p, miR-212-3p and miR-139-5p inhibited RFXAP expression. The results showed miR-212-3p decreased luciferase expression by 37.2%, while the other miRNAs did not significantly decrease luciferase expression (Figure 5B), suggesting that RFXAP is a target mRNA of miR-212-3p.

To confirm PANC-1 derived exosomal miR-212-3p inhibit RFXAP and MHC II in DCs, miR-212-3p mimics and inhibitors were transfected into iDCs and exo-iDCs respectively. Quantitative RT-PCR verified the successful transfection (Figure 5C, 5D). As shown in Figure 5E, RFXAP and MHC II were significantly decreased in inhibitors negative control (NC) transfected exo-iDC than that in mimics NC transfected iDC, which is consistent to figure 4B. miR-212-3p mimics transfected iDCs showed decreased RFXAP and MHC II expression compared with mimics NC transfected iDCs. Inhibitors transfected exo-iDCs showed an increased expression of RFXAP and MHC II compared with inhibitor NC transfected exo-iDC. The results indicated that PANC-1-derived exosomes inhibited RFXAP and MHC II expression via miR-212-3p.

### Pancreatic cancer derived exosomal miR-212-3p inhibited RFXAP and MHC II of iDC

**Figure 6:**
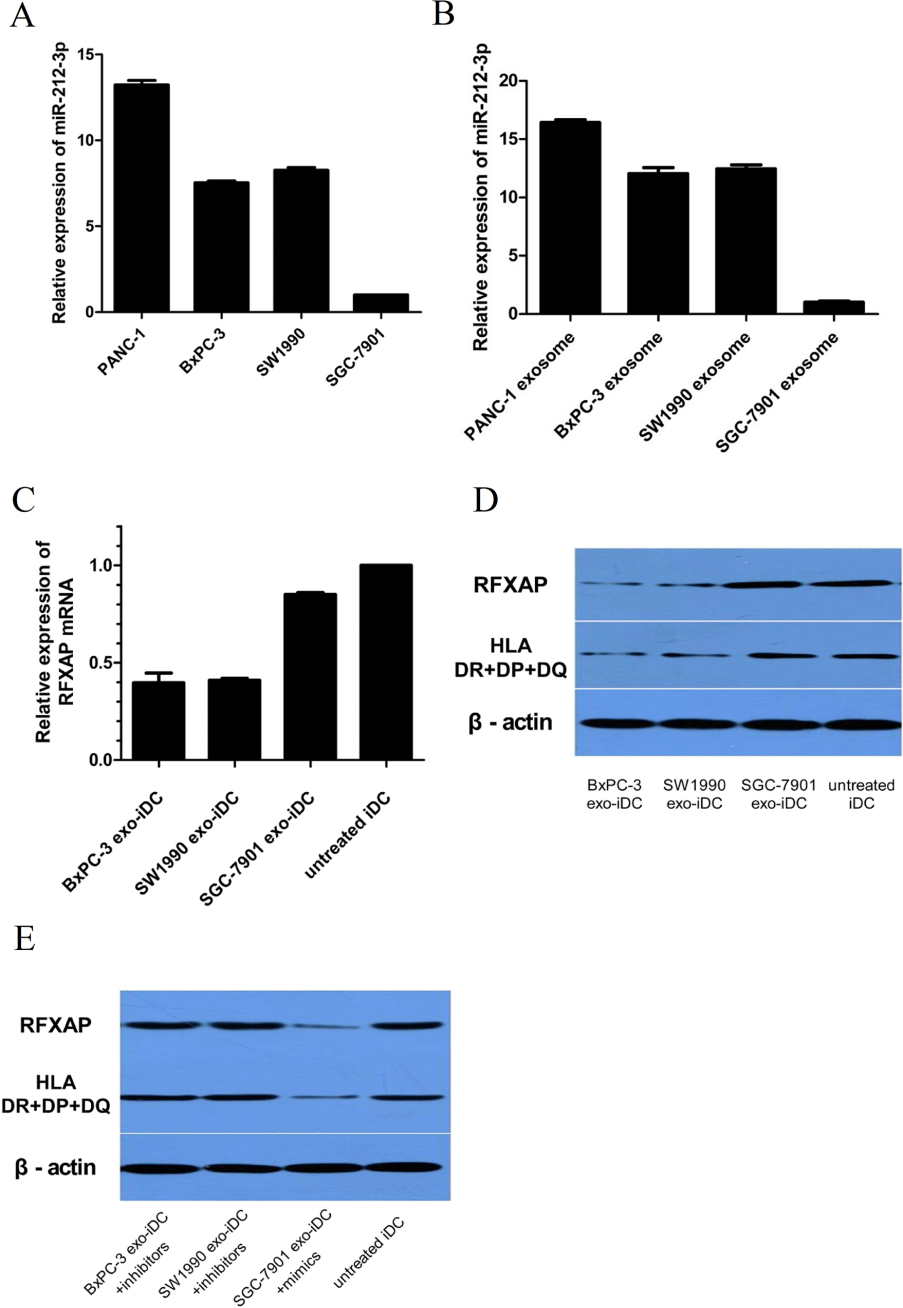
Pancreatic cancer derived exosomal miR-212-3p inhibited RFXAP and MHC II of iDC **A.** qRT-PCR analysis of relative miR-212-3p expression in PDAC cell lines and gastric cancer cell lines. **B.** miR-212-3p expression in tumor cells derived exosome. **C.** qRT-PCR analysis of RFXAP mRNA expression in exosome stimulated iDC. **D.** Western blot analysis of RFXAP and MHC II expression in tumor exosome stimulated iDC. The expression of RFXAP and MHC II were significantly inhibited by SW1990 and BxPC-3 derived exosome, while SGC-7901 exosome did not. **E.** Transfection of miR-212-3p inhibitors and mimics to SW1990, BxPC-3 and SGC-7901 exo-iDCs reversed the expression of RFXAP and MHC II.

To validate if pancreatic cancer derived exosomal miR-212-3p would inhibit RFXAP and MHC II of iDC, iDC were stimulated by SW1990 and BxPC-3 derived exosomes respectively (named as BxPC-3 exo-iDC and SW1990 exo-iDC respectively). It has been confirmed that miR-212-3p were highly expressed in SW1990 and BxPC3 [12], and lowly expressed in a gastric cancer cell line SGC-7901 [13] which was used as negative control in the study. PANC-1, SW1990, BxPC-3 and their exosomes showed higher expression of miR-212-3p than SGC-7901 and its exosomes respectively (Figure 6A, 6B), which were consistent with the previous studies [12, 13]. Compared with untreated iDC, BxPC-3 exo-iDC and SW1990 exo-iDC showed decreased RFXAP and MHC II expression, while SGC-7901 exo-iDC did not decrease significantly. (Figure 6C, 6D).

Then miR-212-3p inhibitors and mimics were transfected to BxPC-3 exo-iDC, SW1990 exo-iDC and SGC-7901 exo-iDC respectively. There were no significant differences of RFXAP and MHC II between inhibitors transfected SW1990 exo-iDC, BxPC-3 exo-iDC and untreated iDC. miR-212-3p mimics transfected SGC-7901 exo-iDCs showed decreased RFXAP and MHC II expression (Figure 6E). The results validated that pancreatic cancer derived exosomal miR-212-3p would inhibit RFXAP and MHC II expression in iDC.

### miR-212-3p was negatively correlated with RFXAP expression in pancreatic cancer

**Figure 7:**
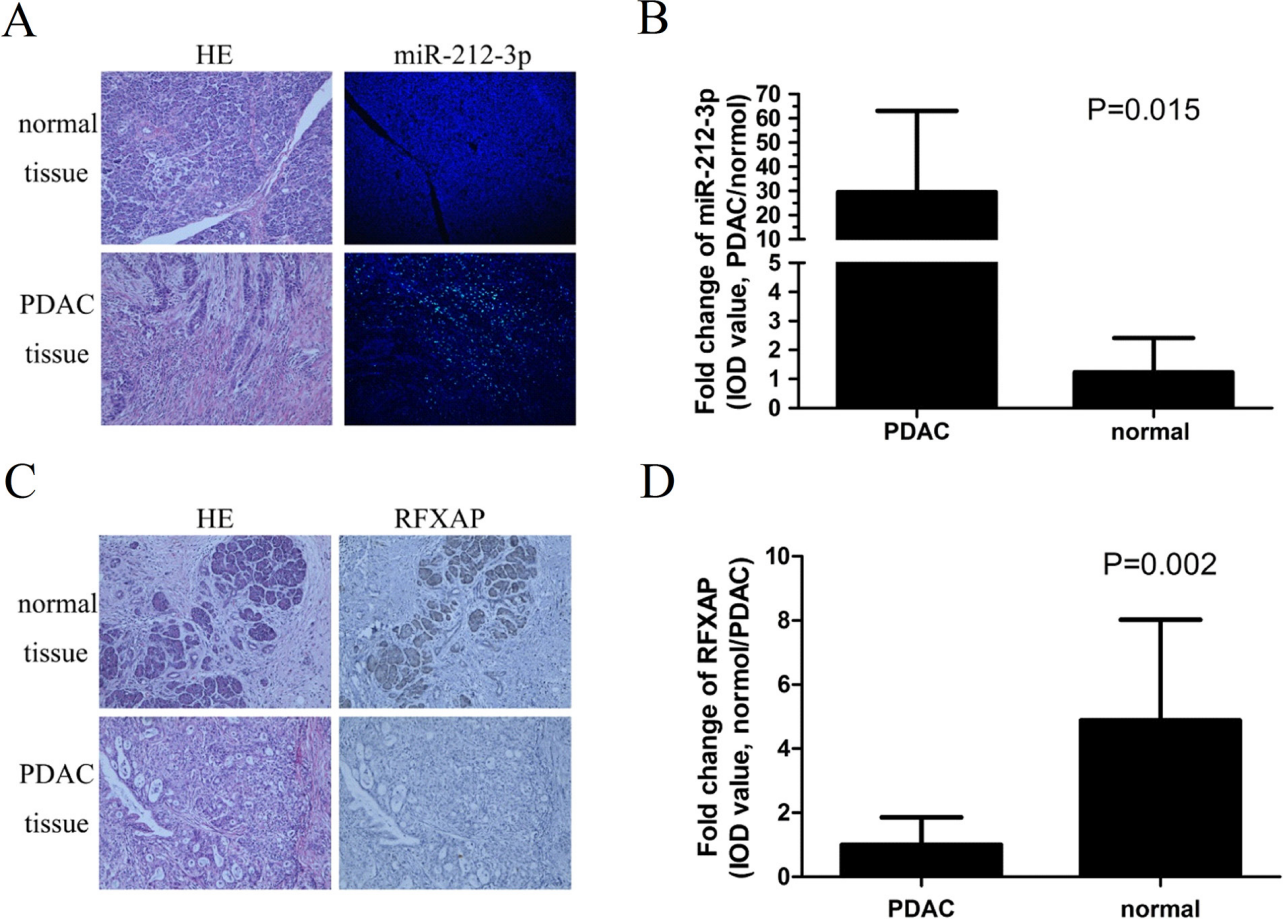
Expression features of miR-212-3p and RFXAP in normal pancreatic tissue and PDAC **A.** HE and FISH of miR-212-3p in normal pancreatic tissue and PDAC. **B.** Comparison of IOD value of miR-212-3p between PDAC and normal pancreatic tissue. The mean IOD of miR-212-3p in PDAC was 29.5 times higher than that of normal pancreatic tissue. Data are presented as means ± S.E.Ms. **C.** HE and IHC staining of the RFXAP in normal pancreatic tissue and PDAC. **D.** The mean IOD of RFXAP in PDAC was 3.8 times lower than that of normal pancreatic tissue. Data are presented as means ± S.E.Ms.

In the clinical PC samples, miR-212-3p and RFXAP expression were examined by fluorescence *in situ* hybridization and immunohistochemistry respectively. miR-212-3p and RFXAP were mainly localized in the cytoplasm and nucleus (Figure 7A, 7C). miR-212-3p was significantly over-expressed in PDAC compared with that in normal pancreatic tissue (*P* < 0.05, Figure 7B), while RFXAP was significantly decreased in PDAC (*P* < 0.05, Figure 7D). By the Pearson correlation test, it was validated that miR-212-3p was significantly negatively correlated with RFXAP in pancreatic cancer (*r* = −0.864, *P* < 0.01).

## DISCUSSION

Since Valadi *et al*. reported exosome-mediated transportation of miRNAs between different cells [14], the mechanisms of exosome-mediated tumor invasion and metastasis have been increasingly studied [15, 16]. Several studies have suggested that tumor exosomal miRNAs are involved in cancer invasion and metastasis by suppressing targeted mRNA in recipient cells [17, 18]. The present study firstly attempted to analyze how the transcriptome of dendritic cells is influenced by PC-derived exosomes, from which we may find valuable information on DC differentiation and function in the microenviroment of pancreatic cancer.

Exosomes containing miRNAs can be transferred into recipient cells by phagocytosis and influence target mRNA expression [19]. It has not been reported that PC-derived exosomes contain tumor-related miRNAs and influence transcriptome expression in DCs. The present study found that 12 out of 84 PC-related miRNAs were highly expressed in exosome-stimulated DCs, including 9 miRNAs which were also the main components of PANC-1 exosomes. This indicated that PC-related miRNAs can be delivered to DCs via exosomes. Furthermore, 208 mRNAs were negatively expressed in exosome-stimulated DCs. The target relationship between increased miRNAs and decreased mRNAs were predicted by searching bioinformatics databases, and the network of potential interaction was portrayed. This is valuable for further studies on how PC-related miRNAs inhibiting mRNAs profile of DCs. From the predicted target network, we found that RFXAP of DCs was inhibited by miR-212-3p transferred from exosome.

Many studies have shown that tumor-derived exosomes induce immune tolerance in tumor microenviroment, leading to tumor invasion and metastasis [20]. Yu *et al* found that tumor-derived exosomes play a crucial role in blocking DC differentiation [21]. However, the mechanisms have not yet been fully elucidated, and deficiency of RFXAP in tumor progression has not been reported previously. RFXAP is a transcription factor for MHC II, and its deficiency leads to bare lymphocyte syndrome, a rare severe immunodeficiency disease caused by MHC II dysfunction [22]. The present study demonstrated that PC-derived exosomes can inhibit RFXAP and MHC II expression via miR-212-3p, which may be an important mechanism of PC-induced immune tolerance.

Gaida *et al*. reported that low expression of MHC II significantly correlated with a worse histological grade of differentiation and relatively poor prognosis in PC [23]. Notably, MHC II molecules are inhibited in most PC [24]. Studies also showed that expression of the transcription factor interferon regulatory factor (IRF) is crucial in the regulation of MHC II expression in DCs, and transfection of the IRF gene can significantly enhance MHC II-mediated antigen presentation [25, 26]. MHC class II negative phenotype of human pancreatic cell lines remains unmodified after treatment with interferon-γ, but transfection of the key genes of the RFX complex resulted in a very high expression of MHC II [27, 28]. The above researches indicated that the deficiency of RFX complex may be the main reason for the low expression of MHC II in PC, although the mechanism behind the dysfunction of the RFX complex has not been demonstrated at the transcriptional level. RFXAP protein is one of the major components of the RFX complex, and its deficiency can lead to a lack of MHC II molecules and immune tolerance [9]. The present study revealed that RFXAP expression was decreased in PC compared with normal pancreatic tissue, which was negatively correlated with high expression of miR-212-3p in PC. RFXAP is inhibited by PC-derived exosomal miR-212-3p, which may be an important mechanism of RFX complex deficiency and MHC II inhibition in PC.

Our study found that miR-212-3p may be a critical factor in PC progression. This is consistent with previous conclusions that miR-212-3p is involved in invasion and metastasis of pancreatic cancer [12, 29]. Similarly, Wu *et al*. revealed that miR-212-3p is crucial in gastric cancer development as it inhibits CD80, an important costimulatory molecule [30].

Pancreatic cancer is one of the most aggressive forms of human cancer, with a 5% 5-year survival rate [31]. Its early invasion and metastasis are of concern for surgeons as they cause a low successful radical resection rate and high post-operative recurrence rate. Therefore, a full understanding of the molecular mechanisms underlying pancreatic tumor progression is essential for improving patient outcome. The present study found that RFXAP deficiency may be a novel mechanism leading to the progression and metastasis of PC. As a transcription factor, RFXAP may also bind other anti-oncogenes, in addition to MHC II. Based on this assumption, it may provide us with new RFXAP binded anti-oncogenes, which may help us to predict early metastasis and develop new target therapy against PC. Of course, further research is still needed to clarify the mechanism of RFXAP in tumor suppression.

In conclusion, we confirmed that PC-related miRNAs can be transferred to DCs via exosomes, leading to inhibition of target mRNAs. The potential target network between PC-related miRNAs and target mRNAs was portrayed, from which we found that RFXAP, an important transcription factor, was inhibited by miR-212-3p. PC-derived exosomes inhibited RFXAP and MHC II expression via miR-212-3p, and this may be a novel mechanism involved in PC invasion and metastasis. Therefore, further studies on RFXAP deficiency in pancreatic cancer may help to find molecular markers related to metastasis and new targets for gene therapy.

## MATERIALS AND METHODS

### Cell culture and exosome isolation

Human pancreatic cancer cell line, including PANC-1, SW1990, BxPC-3 and the gastric cancer cell line SGC-7901 were obtained from Chinese Academy of Sciences (Shanghai, China) and has been tested and authenticated. The cell lines were cultured in RPMI 1640 medium (Hyclone) containing 10% fetal bovine serum (FBS, Gibco, New York, USA) until 90% confluent whereupon the medium was replaced with serum free 1640 medium for 48 h. The cell-free supernatant was spun and supernatants collected from 300 × g for 10 minutes, 2 000 × g for 10 minutes, to remove residual cells and debris, 10 000 × g for 30 minutes to remove microparticles (1), and ultra-centrifuged at 100 000 × *g* for 70 min (Beckman Coulter, California, USA) from which the pellet was resuspended in 50–100 μl PBS. Size distribution within exosome preparations was analyzed by measuring the rate of Brownian motion using a NanoSight (NanoSight NS300, Malvern, UK), which is equipped with a fast video capture and particle-tracking software. Besides, exosomes were allowed to settle on carbon-coated 400-mesh copper grids, stained with 2% uranylacetate, air-dried, and imaged by transmission electron microscopy.

### Induction of DC and stimulation with exosome

Peripheral blood mononuclear cells (PBMCs) were isolated from the venous blood of healthy donors. Briefly, after Ficoll-Paque density gradient centrifugation (Ficoll-Paque, MP Biomedicals, Carlsbad, USA), monocytes were sorted from PBMC using human anti-CD14 microbeads (Miltenyi Biotec, German). The resulting CD14 positive monocyte fraction was cultured for up to 7 days in RPMI 1640 medium, containing 100 U/mL penicillin, 100 ug/ml streptomycin, interleukin-4 (IL-4) (40 ng/mL, Genzyme, Boston, MA, USA) and granulocyte–macrophage colony stimulating factor (GM-CSF) (100 ng/mL, PeproTech, USA). At 7th day, DCs were stimulated by exosome (20 μg per well) for 24 h (exo-iDC), and PBS was used as negative control (iDC). Cells were then centrifuged and used for experiments.

### Profiling of miRNA expression

Total RNA of cells and exosomes was isolated and purified by miRNeasy Mini Kit (Qiagen, Maryland, USA), following the manufacturer's instructions. The concentration, purity, and amount of total RNA were quantified using a NanoDrop spectrophotometer (ND-1000 V3.5.2 software, USA). CDNA was prepared using All-in-one miRNA First Strand cDNA Synthesis Kit (GeneCopoeia, Guangzhou, China). MiProfile™ human pancreatic cancer miRNA qPCR arrays (GeneCopoeia), which contains 84 most abundantly expressed and best characterized human miRNAs associated with pancreatic cancer, was used for expression profile analysis. All-in-one^tm^ qPCR Mix(GeneCopoeia) was used for the real-time PCR reactions on the miRNA PCR Array and the real-time instrument was a ABI 7500 Fast (Applied Biosystems, USA).

### Genome-wide analysis of mRNA transcriptomes

Human genome-wide mRNA microarray (A10201-1-40-56, ribobio, Guangzhou, China) was used for mRNA profiling of iDC and exo-iDC which contains approximately 40 000 transcripts selected from the National Center for Biotechnology Information (NCBI) RefSeq database (Release 56). The mRNAs were the exact same samples used for miRNA expression profile analysis. Briefly, total RNA was amplified and transcribed into fluorescent cRNA by Amino Allyl messageAmp II(Ambion, Life Technologies, Carlsbad, California, USA). Then amplified RNA product was subjected to hybridization, washing, staining and scanning according to the manufacturers’ recommended procedure. The microarray data were deposited in the GEO database under accession number GSE67020.

### miRNA target prediction

Target miRNAs that bound to the candidate mRNAs were predicted by retrieving data bases of MiRanda (http://www.microrna.org/), TargetScan (http://www.targetscan.org/), DIANA microT (http://diana.pcbi.upenn.edu/), miRDB (http://mirdb.org/miRDB/) and miRWalk (http://www.ma.uni-heidelberg.de/apps/zmf/mirwalk/miRWalkis), based on the miRNA and mRNA microarray results.

### Luciferase assay

The 293T cells were co-transfected with pLMP vectors containing *RFXAP* 3′UTR and miRNA mimics. Cells were harvested and subjected to lysis the 48 h after transfection. Renilla luciferase activity was used for normalization, and firefly luciferase activity was detected with a dual luciferase reporter assay kit according to the manufacturer's protocol.

### Transient transfection of DCs with miRNA mimic or miRNA inhibitor

The iDCs and exo-iDCs were transfected with hsa-miR-212-3p mimics and inhibitors (Ribo, Guangzhou, China) respectively. Briefly, iDCs were seeded in 6-well plates. A final concentration of either 50 nM RNA mimics or 100 nM inhibitors RNA was transfected into the cells using lipofectamineTM 2000 (Invitrogen) according to the manufacturer's protocol. The mimics or inhibitors negative control (NC) were used, respectively. 48 hours after transfection, the cells of each group were harvested, followed by PCR and Western blot analysis.

### RNA extraction and qRT-PCR

Total RNA extraction and reverse transcription were performed as mentioned above. PCR was performed by ABI 7500 Fast, including 2 ul of RT product, 10 ul of Power SYBR Green PCR Master Mix (Applied Biosystems, USA), 4 ul of RNase-free water, 2 ul forward and reverse primers. PCR began at 95°C for 10 min, followed by 40 cycles at 95°C for 15 s, 60°C for 1 min. The relative expression ratio of miR-212-3p and RFXAP was presented as the fold change which was normalized to an endogenous reference (U6 RNA). The primers used were purchased from Ribo (Guangzhou, China): hsa-miR-212-3p-F(5′- GGTAACAGTCTCCAGTCA-3′), hsa-miR-212-3p-R(5′-GCAATTGCACTGGATACG-3′), RFXAP-F(5′-CAGTAGAATTCGGCCAAGCAGGTGCTAAAAG-3′), RFXAP-R(5′-CAGAGGATCCATGTAGATGTTCTTGGTAAG-3′).

### Western blot

Exosomes or cells were lysed with RIPA buffer (Sigma, St. Louis, MO, USA) containing a mixture of protease inhibitor cocktail kit (Thermo, Rockford, IL, USA). Then the lysates were cleared by centrifugation and the concentrations of proteins were measured by BCA protein assay kit (Pierce, USA). The proteins were denatured in 2× SDS buffer at 95°C, separated in 10% SDS–polyacrylamide gel electrophoresis (SDS–PAGE) and transferred onto polyvinylidene difluoride (PVDF) membrane (Millipore, Bedford, MA, USA). After being blocked with 5% skim milk powder for 1 h at room temperature, the membranes were probed with the following antibodies: mouse anti-tumor susceptibility gene 101 (TSG101) (1:1 000, Abcam, Cambridge, UK), mouse anti-CD63 (1:1 000, Abcam, Cambridge, UK), mouse anti-Heat Shock Protein-70 (HSP-70, 1:2000, Abcam, Cambridge, UK), RFXAP (1:200, Abcam, Cambridge, UK), or MHC II (1:500, Abcam, Cambridge, UK). The samples were incubated with the secondary goat anti-mouse antibodies conjugated with horseradish peroxidase (1:5 000, Pierce, USA) for 1 h at room temperature. The blots were visualized by enhanced chemiluminescence using Kodak X-OMAT LS film (Eastman Kodak, Rochester, NY).

### Fluorescence *in situ* hybridization (FISH) and immunohistochemistry (IHC) staining

The localization of hsa-miR-212-3p and RFXAP in pancreatic cancer tissue was determined by fluorescence *in situ* hybridization and immunohistochemistry, respectively. In the present study, 20 paired tissues were assessed. All patients provided their written informed consent prior to recruitment.

*In situ* detection was performed using 4-um sections of pancreatic ductal adenocarcinoma tissue with a FITC-labeled LNA oligonucleotide probe for miR-212-3p. The probe was denatured at 73°C and hybridized overnight at 37°C. The procedure was carried out according to manufacturer's instructions. Nuclei were stained with 4,6-diamino-2-phenylindole (DAPI; Sigma, USA). For imaging and processing, a conventional epifluorescence microscope equipped with FITC and DAPI filters (Nikon, Japan) and Image-Pro Plus software was used, respectively.

Immunohistochemical staining was carried out using standard streptavidin-biotin-peroxidase complex method. Briefly, paraffin-embedded tissue sections were dewaxed and rehydrated through an alcohol series, and then endogenous peroxidase activities were blocked. After non-specific sites were saturated with a rabbit polyclonal antibodies(Santa Cruz Biotechnology Inc Santa Cruz, CA, USA), slides were incubated overnight at 4°C with RFXAP antibody (1:100, #ab172281, abcam). Sample incubated with PBS instead of primary antibody were used as negative control.

### Statistical analysis

All data were presented as mean ± standard deviation (SD). and analyzed using student's *t*-test. Pearson correlation was used to analysis the correlation between miR-212-3p and RFXAP expressions. *P* value < 0.05 was considered as statistically significance. All data were processed using SPSS, version 19.0 and Graphpad Prism 5.0 software program.

### Ethics statement

The research protocol was reviewed and approved by the Research Ethics Committee of Sir Run Run Shaw Hospital, School of Medicine, Zhejiang University. All participants or their guardians gave written consent of their tissue samples and medical information to be used for scientific research.

## SUPPLEMENTARY MATERIAL TABLES




