# Supplementary material for: Pancreatic cancer-derived exosomes transfer miRNAs to dendritic cells and inhibit RFXAP expression via miR-212-3p
Source: Oncotarget. 2015 Aug 14;6(30):29877–88. doi: 10.18632/oncotarget.4924 (PMC4745769; doi:10.18632/oncotarget.4924)
Supplement: Supplementary file 1 [file oncotarget-06-29877-s001.pdf]

SUPPLEMENTARY TABLES

Supplementary Table S1: fold change of gene expression between exosome-stimulated DCs (exo-iDC) and untreated DCs (iDC)

Supplementary Table S2: MiRNA-mRNA interaction network

miR-188-5p	miR-203	miR-202-3p	miR-101-3p	miR-139-5p	miR-212-3p	miR-210	miR-424-5p	miR-9-5p
ANKRD29	AHCTF1	AHCTF1	ARHG AP11A	BBS7	ANKRD29	ANKRD29	AHCTF1	ARHG AP11A
ARHG AP11A	ANKRD29	BTF3L4	BBS7	BTF3L4	ARHG AP11A	EPB41L5	ANKRD29	AS3MT
BOLA1	ARHG AP11A	CAMK2D	C18orf32	CAMK2D	BBS7	ETV1	ARHG AP11A	BBS7
BTF3L4	BBS7	CC2D2A	CAMK2D	CC2D2A	CAMK2D	FANCF	BTF3L4	BTF3L4
C11orf63	BTF3L4	CREM	CREM	CREM	CC2D2A	GPD2	CAMK2D	CCDC140
CC2D2A	C11orf63	CXorf23	CXCL11	CXCL11	CXorf23	MICAL3	CC2D2A	CHMP7
DNAJB4	C18orf32	DCN	CXorf23	CXorf23	DST	NFAT5	CCDC83	CREM
DST	CAMK2D	DKK1	DKK1	DCN	EID2B	NLGN4Y	COPS2	CXCL11
EFNA1	CCDC83	DST	DST	DNAJB4	EPB41L5	NUFIP2	CREM	DST
EIF2AK3	CFH	DUSP1	DUSP1	DST	ETV1	PRDX2	CXorf23	DUSP1
EPB41L5	CHMP7	DYNC2LI1	DYNC2LI1	DUSP1	EXOC1	RNF149	DNAJB4	EFNA1
ETV1	CREM	EIF2AK3	EFNA1	DYN C2LI1	FAM63A	SEC24B	DST	EID2B
FAM63A	CXCL11	EPB41L5	EPB41L5	EIF2AK3	FCGR2B	SLC8A3	EIF2AK3	FAM63A
FXYD4	CXorf23	GIPC2	ETV1	EPB41L5	FXYD4	VASP	EPB41L5	FANCF
GAS2L2	DBR1	KANK1	EXOC1	EXOC1	GPD2		ETV1	GPD2
GIPC2	DCN	KCNG3	FAM63A	FAM63A	KCNG3		EXOC1	KANK1
GPD2	DKK1	LDHAL6B	GPD2	GPD2	MICAL3		FAM63A	KLF4
KCNG3	DNAJB4	MID1	KANK1	IFT81	MOSPD1		GIPC2	MEFV
MEFV	DST	NFAT5	KCNG3	KCNG3	NFAT5		GPD2	MICAL3
MICAL3	DUSP1	NRCAM	MEFV	MBD2	NLGN4Y		KANK1	MID1
MOSPD1	DYNC2LI1	OPRM1	MGST1	MESP2	NRCAM		KCNG3	NLGN4Y
NFAT5	EID2B	PRDM11	MID1	MICAL3	NT5C3		KLF4	NRCAM
NRCAM	EPB41L5	RPGRIP1L	MOSPD1	MOSPD1	NUFIP2		MEFV	NT5C3
NUDCD1	ETV1	SDK1	NFAT5	NFAT5	PDK1		MESP2	NUDCD1
PDK1	FCGR2B	TFAP2A	NLGN4Y	NLGN4Y	PRDX2		MICAL3	NUFIP2
PTGER4	GIPC2	TUSC3	NOX1	NOX1	PTGER4		MID1	OPRM1
PTPLAD2	GIPR		NRCAM	NRCAM	RFXAP		NFAT5	PDK1
RPGRIP1L	GPD2		NUDCD1	NUFIP2	RPGRIP1L		NUFIP2	PRDM11
SDK1	KANK1		NUFIP2	OPRM1	SETD3		PDK1	PTPLAD2

miR-188-5p	miR-203	miR-202-3p	miR-101-3p	miR-139-5p	miR-212-3p	miR-210	miR-424-5p	miR-9-5p
SLC8A3	KCNG3		PRDX2	PDK1	SLC8A3		PTGER4	RPAP3
TRMT11	MEFV		PTGER4	PIWIL2	SLITRK5		RNF149	RPGRIP1L
VASP	MGST1		RFXAP	PTPLAD2	TMEM64		RPAP3	SDK1
	MICAL3		RPGRIP1L	RFXAP	TRAT1		RPGRIP1L	SETD3
	MID1		SEC24B	RNF149	TRIM23		SDK1	SLC8A3
	MOSPD1		SETD3	SCHIP1	TUSC3		SEC24B	SMYD3
	NFAT5		SLITRK5	SDK1			SETD3	TRAT1
	NLGN4Y		TFAP2A	SETD3			TFAP2A	
	NRCAM		TMEM64	SLITRK5			TRIM23	
	NUDCD1		TRIM23	TRIM23			VASP	
	NUFIP2			TUSC3				
	PDK1			ZNF555				
	PLEKHG5							
	PRDX2							
	PTGER4							
	PTPLAD2							
	RFXAP							
	RNF149							
	RPGRIP1L							
	SCHIP1							
	SDK1							
	SEC24B							
	SETD3							
	SLITRK5							
	TMEM64							
	TRAT1							
	TRIM23							
	TUSC3							
	VASP							
	ZNF555							
